# Supplementary figures and images for: Lactate-induced histone lactylation by p300 promotes osteoblast differentiation
Source: PLoS One. 2023 Dec 5;18(12):e0293676. doi: 10.1371/journal.pone.0293676 (PMC10697613; doi:10.1371/journal.pone.0293676)

**Fig. S1.**  
**Inhibition of LDH was not changed histone acetylation levels in C2C12 cells.**

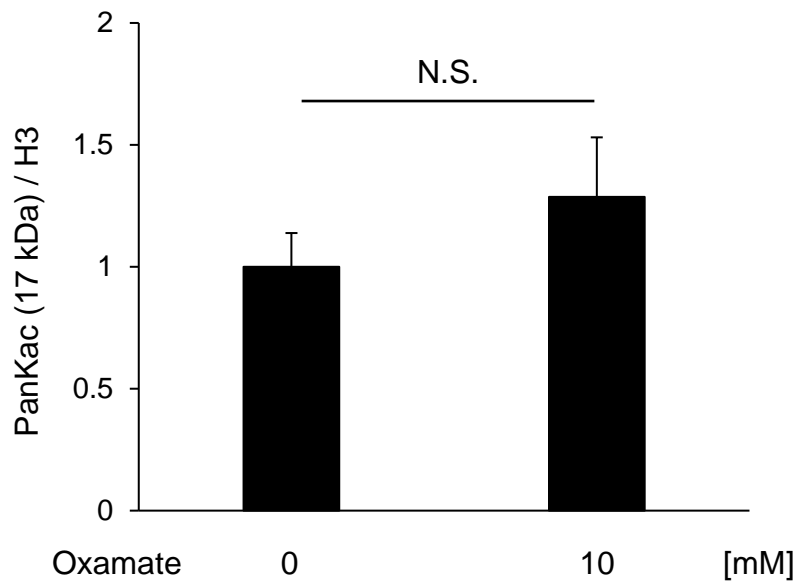

Supplement: S1 Fig — C2C12 cells were incubated for 24 hours after addition of BMP-2 (150 ng/ml) with Oxamate. Amounts of expression of acetyl lysine after addition of BMP-2 and oxamate was evaluated by western blotting. Data are expressed as mean ± SD (n = 3). *, **Significantly different from control group (*p <0.05, **p <0.01). (PDF) [file pone.0293676.s001.pdf]

**Fig. S2.**  
*Ep300* siRNA decreased expression of *Ep300* mRNA in C2C12 cells.

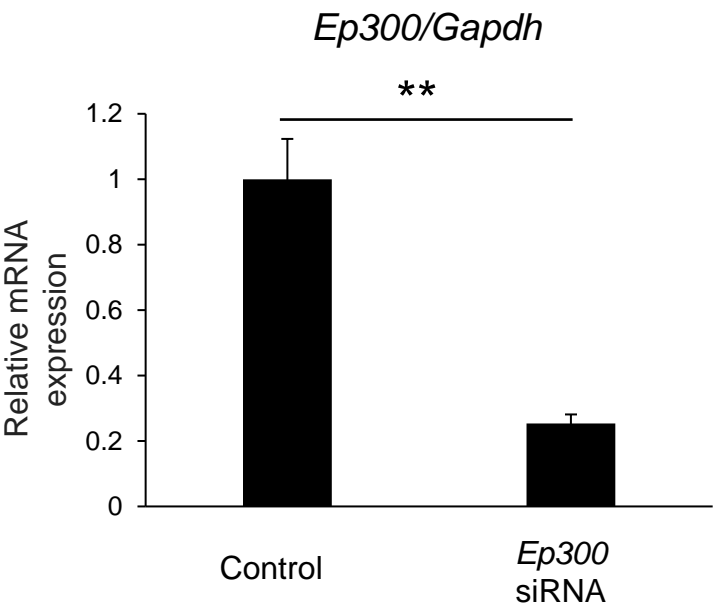

Supplement: S2 Fig — Following introduction of Ep300 siRNA, C2C12 cells were cultured for 72 hours after addition of BMP-2 to the medium. Expression of mRNA for Ep300 were analyzed by real-time PCR. Amplification signals from the gene was normalized that of Gapdh. Data are expressed as mean ± SD (n = 3). *, **Significantly different from control group (*p <0.05, **p <0.01). (PDF) [file pone.0293676.s002.pdf]

**Fig. S3.**

***Ep300* siRNA decreased histone acetylation levels in C2C12 cells.**

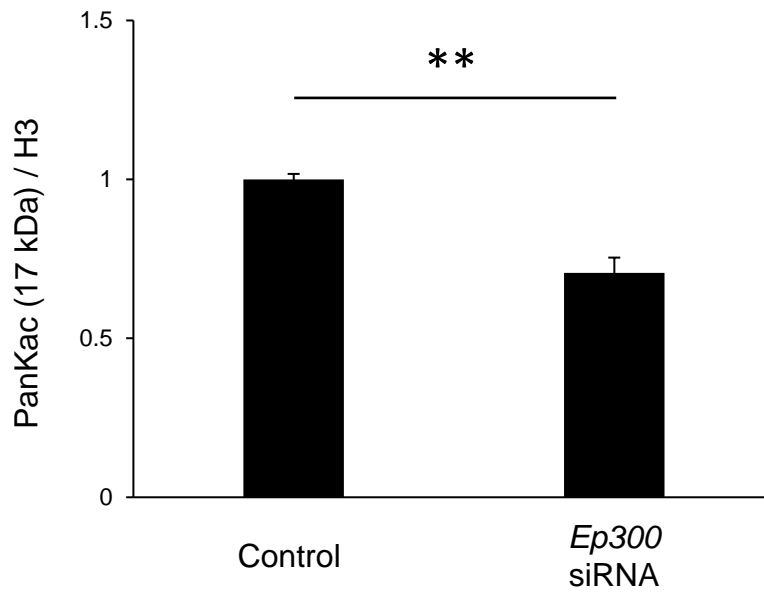

Supplement: S3 Fig — Following introduction of Ep300 siRNA, C2C12 cells were cultured for 24 hours after addition of BMP-2 to the medium. Amounts of expression of acetyl lysine was evaluated by western blotting. Data are expressed as mean ± SD (n = 3). *, **Significantly different from control group (*p <0.05, **p <0.01). (PDF) [file pone.0293676.s003.pdf]
