# Supplementary material for: Lactate-induced histone lactylation by p300 promotes osteoblast differentiation
Source: PLoS One. 2023 Dec 5;18(12):e0293676. doi: 10.1371/journal.pone.0293676 (PMC10697613; doi:10.1371/journal.pone.0293676)
Supplement: S1 Raw images — (PDF) [file pone.0293676.s004.pdf]

Fig.1 d

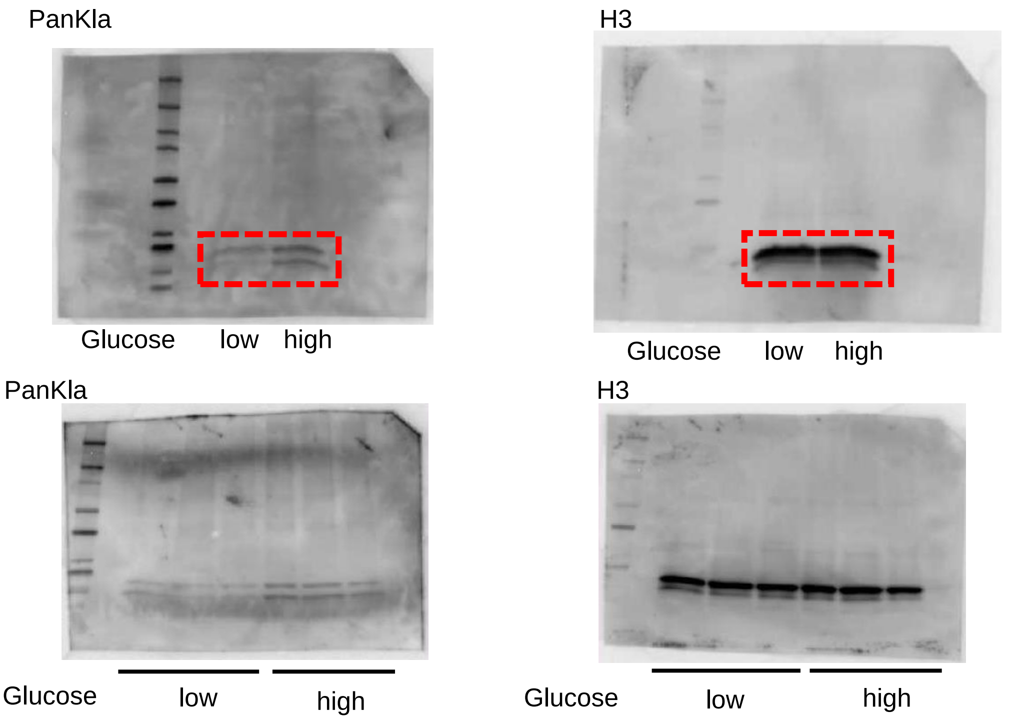
PanKla
H3
Glucose low high
Glucose low high
PanKla
H3
Glucose low high
Glucose low high

Fig.2 d

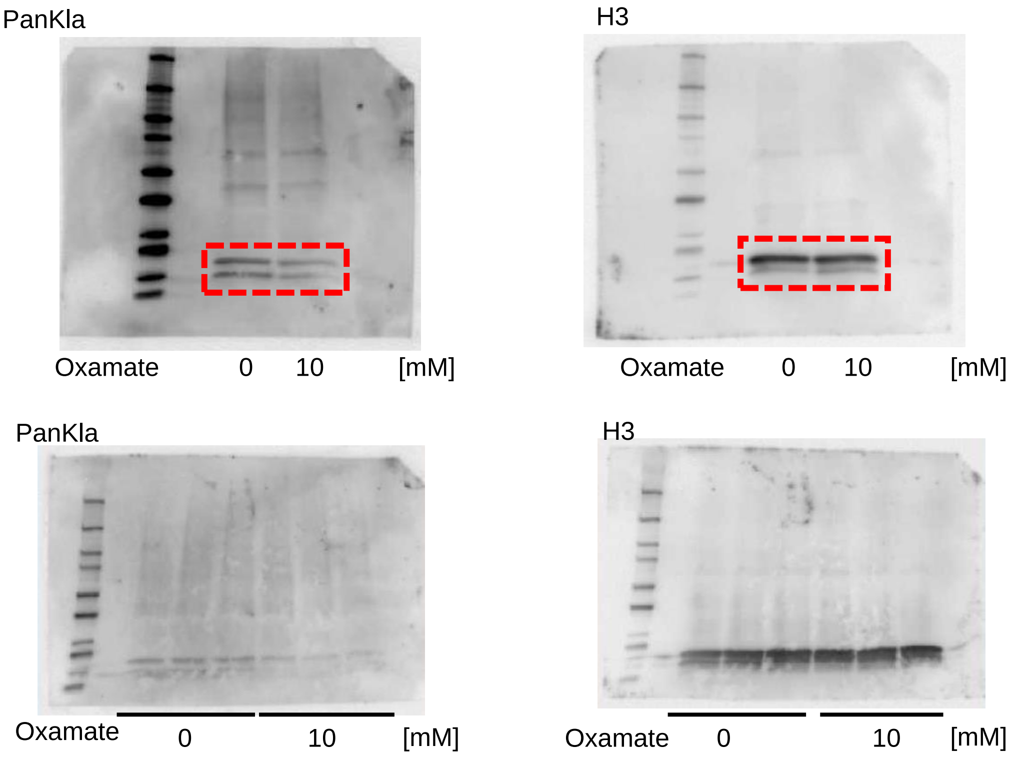
PanKla
H3
Oxamate 0 10 [mM]
Oxamate 0 10 [mM]
PanKla
H3
Oxamate 0 10 [mM]
Oxamate 0 10 [mM]

Fig.3 d

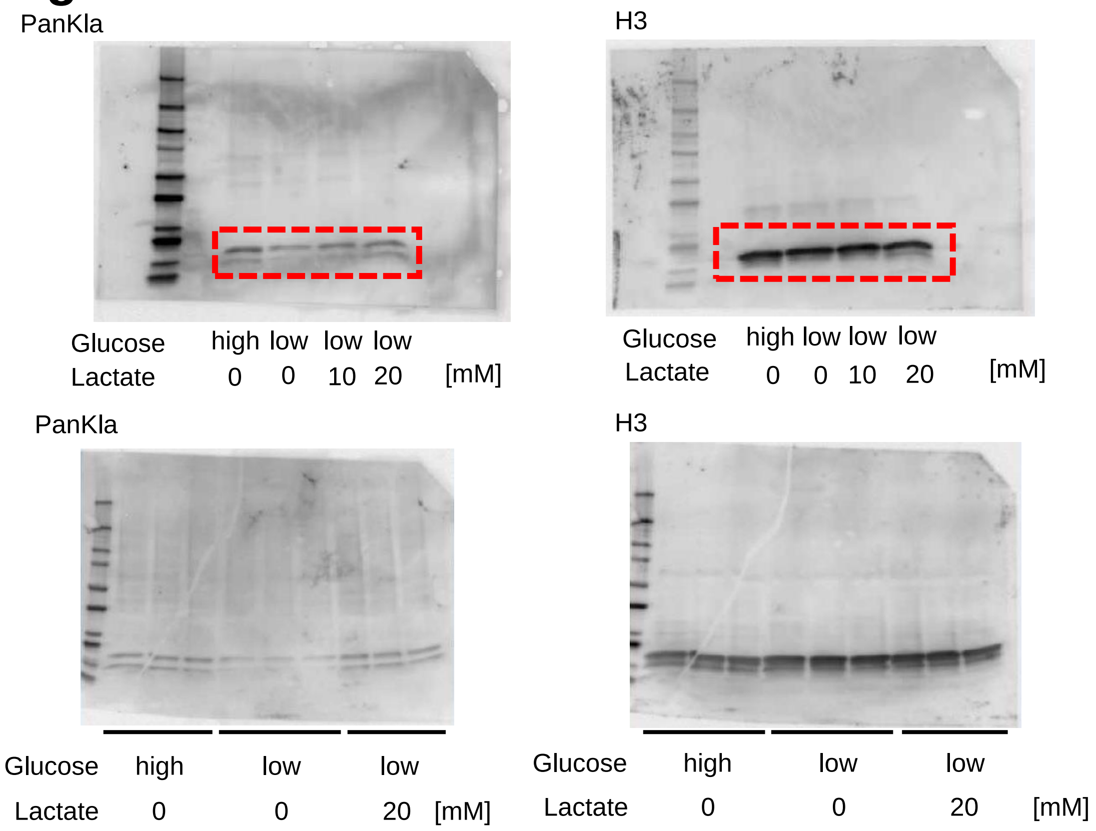
PanKla
H3
Glucose high low low low
Lactate 0 0 10 20 [mM]
Glucose high low low low
Lactate 0 0 10 20 [mM]
PanKla
H3
Glucose high low low
Lactate 0 0 20 [mM]
Glucose high low low
Lactate 0 0 20 [mM]

Fig.4 d

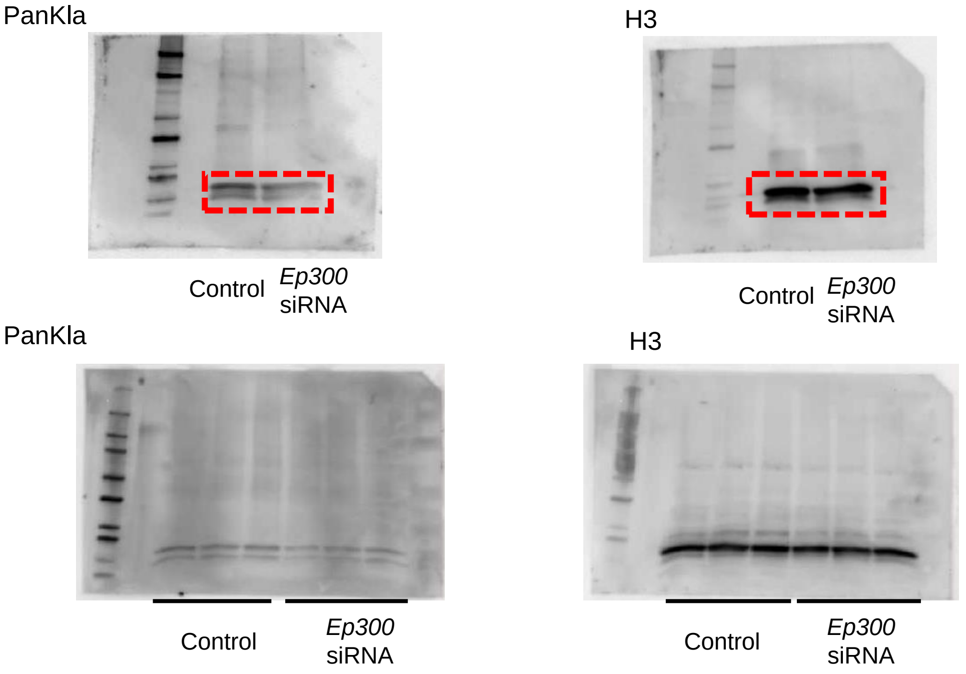
PanKla
H3
Control *Ep300* siRNA
Control *Ep300* siRNA
PanKla
H3
Control *Ep300* siRNA
Control *Ep300* siRNA

Fig. S1

PanKac

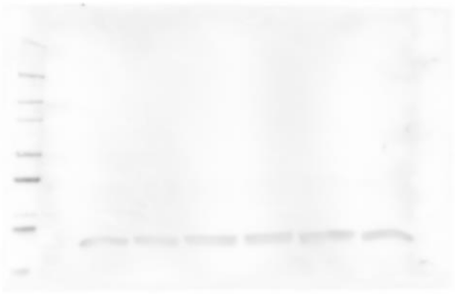

Oxamate 0 10 [mM]

H3

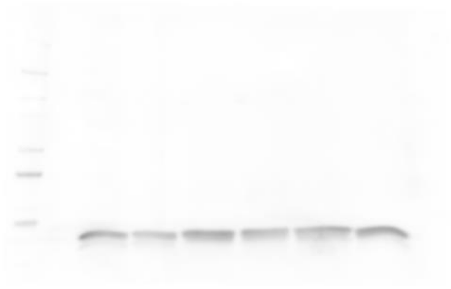

Oxamate 0 10 [mM]

Fig. S3

PanKac

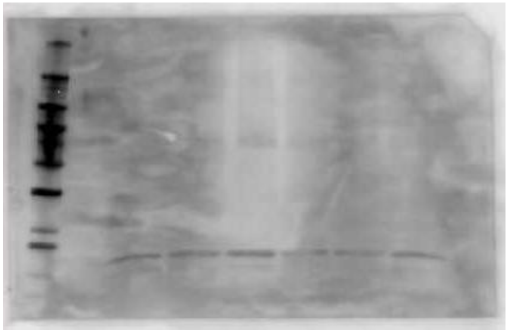

Control

Ep300 siRNA
